# Comparison of INTAKE24 (an Online 24-h Dietary Recall Tool) with Interviewer-Led 24-h Recall in 11–24 Year-Old

**DOI:** 10.3390/nu8060358

**Published:** 2016-06-09

**Authors:** Jennifer Bradley, Emma Simpson, Ivan Poliakov, John N. S. Matthews, Patrick Olivier, Ashley J. Adamson, Emma Foster

**Affiliations:** 1 Institute of Health and Society, Newcastle University, Newcastle upon Tyne NE2 4AX, UK; Jen.bradley@ncl.ac.uk (J.B.); Emma.simpson@ncl.ac.uk (E.S.); Ashley.adamson@ncl.ac.uk (A.J.A.); 2 Human Nutrition Research Centre, Newcastle University, Newcastle upon Tyne NE2 4HH, UK; 3 Open Lab, School of Computing Science, Newcastle University, Newcastle upon Tyne NE1 8HW, UK; Ivan.poliakov@ncl.ac.uk (I.P.); Patrick.olivier@ncl.ac.uk (P.O.); 4 School of Mathematics and Statistics, Newcastle University, Newcastle upon Tyne NE1 7RU, UK; John.matthews@ncl.ac.uk

**Keywords:** INTAKE24, 24-h dietary recall, interviewer-led 24-h dietary recall, automated multiple-pass method, AMPM, dietary assessment methods, online dietary assessment tools

## Abstract

Online dietary assessment tools offer a convenient, low cost alternative to traditional dietary assessment methods such as weighed records and face-to-face interviewer-led 24-h recalls. INTAKE24 is an online multiple pass 24-h recall tool developed for use with 11–24 year-old. The aim of the study was to undertake a comparison of INTAKE24 (the test method) with interviewer-led multiple pass 24-h recalls (the comparison method) in 180 people aged 11–24 years. Each participant completed both an INTAKE24 24-h recall and an interviewer-led 24-h recall on the same day on four occasions over a one-month period. The daily energy and nutrient intakes reported in INTAKE24 were compared to those reported in the interviewer-led recall. Mean intakes reported using INTAKE24 were similar to the intakes reported in the interviewer-led recall for energy and macronutrients. INTAKE24 was found to underestimate energy intake by 1% on average compared to the interviewer-led recall with the limits of agreement ranging from minus 49% to plus 93%. Mean intakes of all macronutrients and micronutrients (except non-milk extrinsic sugars) were within 4% of the interviewer-led recall. Dietary assessment that utilises technology may offer a viable alternative and be more engaging than paper based methods, particularly for children and young adults.

## 1. Introduction

Online dietary assessment tools are an increasingly popular choice for large-scale dietary surveys. They provide a solution for many problems encountered with traditional “gold standard” dietary assessment methods such as weighed food records (WFR). WFR can impose a high level of participant burden resulting in poor completion rates, and can be costly to run [1]. Web-delivered dietary assessment methods offer the potential to be significantly more convenient for participants as they can be completed at times and places that are suitable for them. They also substantially reduce the cost of nutritional analysis, which can be performed without the need for (or with minimal) manual data entry and coding. Online methods also ensure consistency of coding, and offer the potential to make dietary assessment more intuitive and engaging for the user. As with all dietary assessment methods there are limitations to digital tools. Although extensive food lists are created and regularly updated, it is possible that some foods will be missing from a system database. This is especially likely for the diets of minority ethnic groups [2]. There is also evidence that digital systems underestimate nutrient intakes compared to traditional methods [3,4].

INTAKE24 is an online multiple pass 24-h dietary recall tool developed by a team of nutritionists and computer scientists at Newcastle University, for use in a national food and nutrition survey of 11–24 year-old. The system was created and refined through an iterative process that involved four cycles of user study, evaluation and system development [5]. Evaluation focused primarily on the usability of the system (e.g., how easy it is to learn and use) and user experience (e.g., how satisfying, enjoyable and motivating the system is to use) [6].

The potential convenience and the low-cost nature of online dietary assessment has led to a growing number of such systems being created for use in dietary surveys [4,7,8]. One of the first systems to be developed was ASA24 (Automated Self-Administered 24-h Recall), designed by Subar and colleagues to be a low-cost system which is easy to use [9]. The tool is based on the United States Department of Agriculture (USDA) Automated Multiple Pass Method (AMPM) [10], and has been validated in a feeding study with 81 adults [11]. A version has also been produced for use with children, ASA24-Kids, and validated against school lunch observations [12]. Further details on both systems and demos are available online [13]. Myfood24 is an online dietary assessment system which has been developed for use with the UK population and has been validated against face-to-face interviewer-led recalls in 11–18 years old [7,14]. INTAKE24, ASA24 and myfood24 are all based on the principles of the AMPM method, however myfood24 only adopts some of these aspects.

The objective of the present study was to undertake a comparison of INTAKE24 (the test method) with interviewer-led multiple pass 24-h recalls (the comparison method) in 180 people aged 11–24 years (60 participants aged 11–16 years and 120 aged 17–24 years). The age range of 11–24 years was selected, as the system had previously been developed and tested with 11 to 16 years old, and Food Standards Agency Scotland wanted to test the system with this age group and young adults. We acknowledge that this is a wide age range likely to differ in both their food knowledge and ability and motivation to report their dietary intake.

INTAKE24 was compared against an established, widely used face-to-face method, previously used in the Low Income Diet and Nutrition Survey (LIDNS) in the UK [15,16]. The LIDNS recall method is based on the multiple pass method used by USDA [10] and has been validated against direct observation with 42 men aged 21–65 years [17] and doubly labelled water with 524 volunteers aged 30–69 years [18]. The method was tested in a comparison study where participants (*n* = 384) aged 2–90 years old completed 4 recalls, 4-day weighed diaries, 4-day semi-weighed food diaries and a food checklist [19].

## 2. Materials and Methods

Ethical approval for the study was granted by the Newcastle University Faculty of Medical Sciences Ethics Committee (00706/2013).

### 2.1. Recruitment

The recruitment of 11–16 year-old was conducted by the research team at Newcastle University. Participants were recruited from secondary schools in Dundee and Newcastle upon Tyne. The 17–24 year-old were initially recruited by a recruitment agency who approached potential participants on the street. However, because there was a time delay of approximately four weeks between recruitment and the start of the study, the drop-out rate was very high (61%). Therefore, to reach the target sample of 120 17–24 year-old, the final sample of participants was recruited by both the agency and the research team. The research team boosted recruitment by 39 17–24 years old through posters, email advertisements and snowballing techniques.

All participants were required to give written consent (parental consent obtained for those under the age of 18) before participating in the study. The method of recruitment was the same for both age groups; all participants were given an information sheet explaining the study, and if they were interested in taking part, they were asked to complete a consent form. Quotas were used to ensure a representative sample of participants was recruited. The quotas ensured an even distribution in age and gender, and a representative sample in terms of economic status (higher education, working, unemployed, at school, or looking after home/family) and ethnicity (white or non-white). These categories were similar to those used in previous Scottish national surveys [20].

### 2.2. Data Collection

Data collection took place between December 2013 and March 2014. Each participant was asked to complete INTAKE24 and an interviewer-led 24-h recall on the same day on four separate days (including at least one weekend day) over a one-month period. Participants were asked to recall all foods and drinks consumed the previous day (from midnight to midnight). Practical and financial constraints meant use of an objective measure such as doubly labelled water or a feeding study was not possible. As the process of completing the first recall is likely to enhance the accuracy of the second recall a weighted randomisation was used whereby 75% of participants completed INTAKE24 first on each occasion, and 25% completed the interviewer-led recall first on each occasion. Testing the online recall after completing the interviewer-led recall is testing the system in a way in which it would never be used in practice. Completing INTAKE24 first ensures the best possible quality of interviewer-led recall (comparison method); that is, a recall enhanced by having completed the online recall first, allowing us to be as critical as possible of INTAKE24. Asking a subsample to complete the interviewer-led recall first, acts as a methodological check to estimate the impact of completing this recall first, on the accuracy of the INTAKE24 recall (completed second).

The researcher took participant height and weight measurements unless the participant declined to be measured or the meeting place was not suitable, for example café, library, *etc*.

### 2.3. INTAKE24

**Figure 1:**
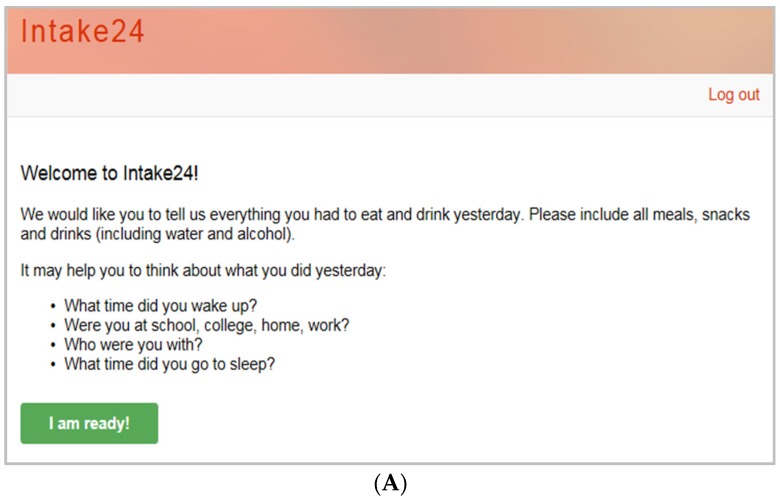

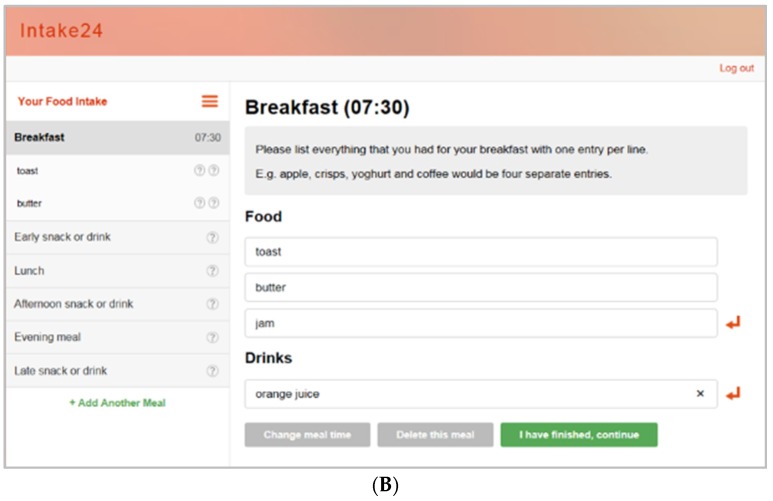
Screen shots of INTAKE24; (**A**) User instructions for INTAKE24; (**B**) Food and drink entry interface.

**Figure 2:**
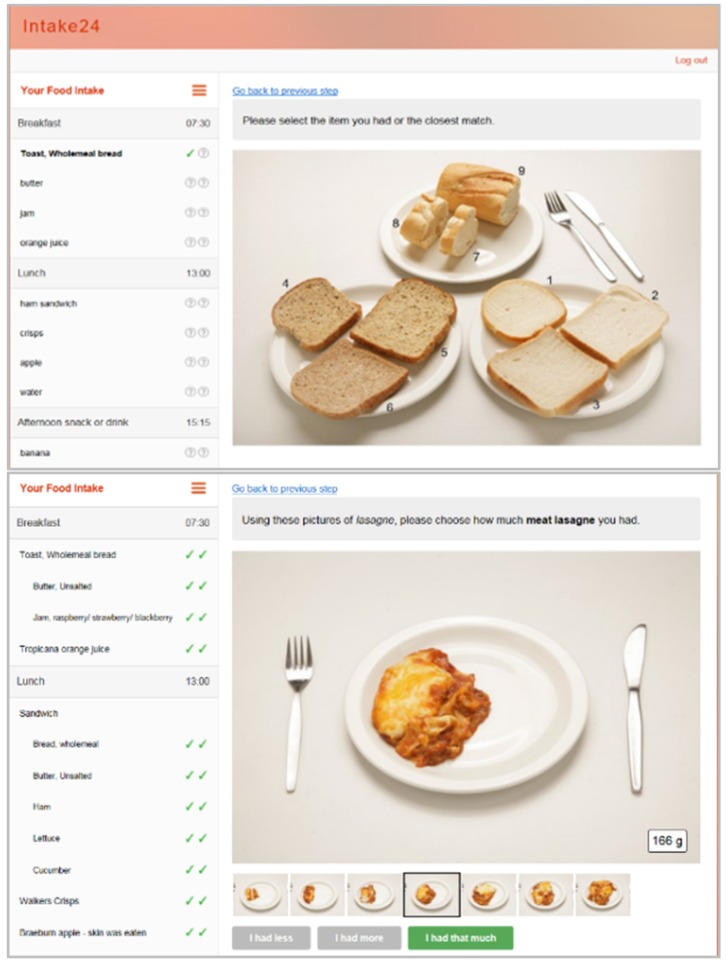
Examples of food portion estimation methods in INTAKE24.

The user begins by entering all foods and drinks consumed the previous day (from midnight to midnight) using free text entry (Figure 1); these are matched to foods within the system database. The user then estimates portion size using a series of over 3000 food photographs (Figure 2). These have been developed based on the portion sizes of foods reported in the UK National Diet and Nutrition Surveys (NDNS), and have been extensively validated in both a feeding study and a relative validation against 4-day weighed intakes [21]. Foods within the system are linked to the NDNS Nutrient Databank and all data are automatically coded. Further details on the system and a demo are available online [22]. 

### 2.4. Interviewer-Led Recalls

The interviewer-led recalls followed the same interview protocol used in LIDNS [15]. All interviews were completed by five researchers with a nutrition background, who were trained by the Principal Investigator to follow a standard script and were observed during the data collection period to ensure consistency of the interview process. Portion size assessment was assisted by the use of the Young Persons Food Atlas [23]. For practical reasons the interviews were scheduled with the participants in advance, the day and time of interview needed to be agreeable for the participant and therefore the time of day and the number of days between recalls varied.

### 2.5. Data Collection: 11–16 Year-Old

All data collection for 11–16 year-old took place in the food technology departments of two schools; one in Dundee and one in Newcastle upon Tyne. Researchers were based in schools for a total of 21 days.

Each participant was issued with a unique username and password and provided with the URL (*i.e.*, web address) with which they could access INTAKE24. They were asked to follow the on-screen instructions and complete INTAKE24 unassisted. Each participant was asked to complete the recall in one sitting, and given as much time as they needed. They were not observed by researchers. Once the participant had completed the online recall, they immediately completed the 24-h recall with an interviewer (vice-versa for those completing the interviewer-led recall first). Each participant was offered an incentive of high street shopping vouchers to the value of £15 on completion of all four pairs of dietary recalls (INTAKE24 and interviewer-led).

### 2.6. Data Collection: 17–24 Year-Old

For the 17–24 years old the first dietary recall interviews took place in person, either at the participant’s home or a place convenient for them; for example, a university, library, or café.

For participants who completed INTAKE24 first, an email was sent the day before the appointment to confirm the meeting and to provide the URL (as a hyperlink) and login details for the online system. The email stressed that they must complete the online recall on the day of the researcher’s visit in advance of the interviewer-led recall. A text message was also sent on the morning of the appointment to remind participants to complete INTAKE24.

For participants who completed the interviewer-led recall first, the URL for INTAKE24 was sent, via email, to the participant once they had completed the face-to-face interview. It was stressed that the online recall had to be completed later that day. Researchers logged onto the administration pages of INTAKE24 to check completion, and if no survey had been submitted later that day, a reminder text message was sent to the participant. Participants were not able to re-access their INTAKE24 recall after submission.

On completion of the interviewer-led recall, the researcher explained that the remaining interviewer-led recalls would be completed by telephone. This is the method used in the LIDNS and only minor differences were found between the modes of administration [24]. A Young Persons Food Atlas [23] was left with the participant, along with a stamped addressed envelope to return the book once they had finished the study. An incentive in the form of high street shopping vouchers of the value of £30 were offered to 17–24 year-old on completion of all four sets of dietary recalls. Participants were advised that they would receive their incentive only once the food atlas had been returned.

**Figure 3:**
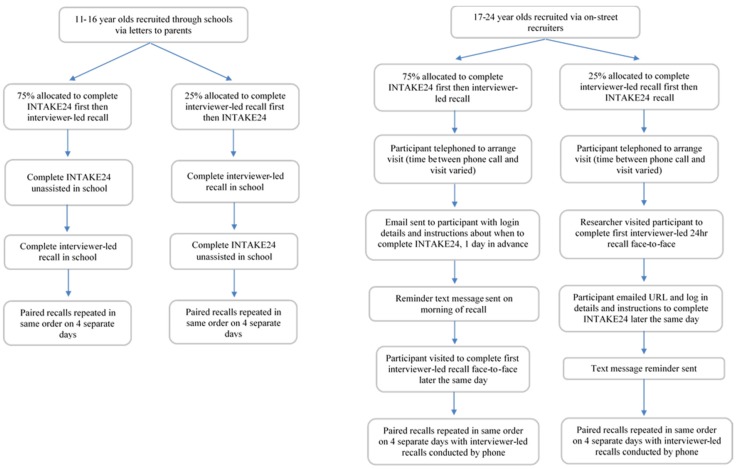
Flow chart detailing participant flow through the study.

A flow chart summarising the study design is given in Figure 3.

### 2.7. Data Entry

INTAKE24 automatically codes the recall and provides nutritional output. Foods contained in the system are linked to NDNS databank food codes and portion size images are linked to a database of weights. If the participants could not find the food they required when using INTAKE24 (either because of failure to locate the food or because the food was missing from the system database) they were asked to select the closest match. Analysis of the INTAKE24 database identified 77 food search terms (1% of the total), which had resulted in a selection of a “closest match” and these foods were subsequently added to the database. Examples include “protein shake” coded as “milk shake made with powder”, “almond milk” coded as “semi skimmed milk” and “chicken curry pie” coded as “chicken curry ready meal”.

The interviewer-led recalls required manual coding using NDNS databank food codes and the data was entered into a purpose-built database. Coding was completed by 2 researchers trained to Bachelor level in nutrition and 20% of the data were manually checked. The INTAKE24 and the interviewer-led recall datasets were merged.

### 2.8. Statistical Analysis

Mean daily intakes were analysed for all participants completing any number of days, *i.e.*, at least one INTAKE24 and one interview-led recall on the same day. Unpaired recalls, where the participant had completed only INTAKE24 or the interviewer-led recall for that day, were not included (*n* = 17).

The impact of errors in reported nutrient intakes was investigated by calculating the ratio of an individual’s daily energy and nutrient intakes based on the INTAKE24 recall to their daily energy and nutrient intakes reported in the corresponding interviewer-led recall, for each day recorded. The Bland and Altman method was used to look at the limits of agreement of the two methods [25]. Limits of agreement are applied so that 95% of the differences will lie between the limits, this is calculated by Equation (1):

Limits of agreement *= d* ± 2*s* (1)

where *d* = mean difference, *s* = standard deviation of the differences. As the data were not normally distributed the analyses were performed on the logged weights of the foods and nutrients. The log of the ratio of the weights is equal to the difference between the log of the weights (*i.e.*, log of (fat (g) by INTAKE24: fat (g) by interviewer-led recall) is the same as (log of fat (g) by INTAKE24) minus (log of fat (g) by interviewer-led recall)). The values presented are the ratio of the geometric mean. The effect of the order of administration was analyzed by comparing the ratio of the geometric mean and the standard deviation for those completing INTAKE24 first with those completing interviewer-led recall first.

Analysis by food groups was also conducted. Each food consumed was assigned to a food group; these food groups were agreed during discussions with the project funders.

## 3. Results

### 3.1. Completion Rates

A total of 168 participants completed at least one corresponding INTAKE24 and interviewer-led recall (completing both INTAKE24 and the interviewer-led recall on the same day) (Table 1). 149 participants completed three or more corresponding recalls; 48 11–16 year-old and 101 17–24 year-old. 129 participants completed all four corresponding recalls; 45 11–16 years old and 84 17–24 years old.

### 3.2. Agreement between INTAKE24 and Interviewer-Led Recalls

Mean ratios were calculated by dividing the nutrient reported using INTAKE24 by the intake of that nutrient reported during the interviewer-led recall. Limits of agreement were applied so that 95% of the differences in intakes would lie between the limits. INTAKE24 was found to provide estimates of energy intake that were 3% lower on average than the interviewer-led recall for the younger age group, with the limits of agreement ranging from minus 48% to plus 82%. Mean intakes of all macronutrients and micronutrients were within 10% of the interviewer-led recall (Table 2). For the older age group, estimates of energy intake were in agreement on average for both methods, with limits of agreement ranging from minus 50% to plus 98%. Mean intakes of all macronutrients and micronutrients were within 3% of the interviewer-led recall with the exception of alcohol which was estimated to be 13% lower on average using INTAKE24 compared with the interviewer-led recall (Table 3). Our study sample reported very low intakes of alcohol on average using both methods and the distribution of intake of alcohol was skewed with only a small number of people reporting consuming alcohol on the recalled days.

There was little difference between the two age groups in terms of agreement between the two methods. Exceptions were seen for some nutrients: fat (0.92 and 0.99 for the younger and older age group respectively), NSP (0.94 and 1.02 for the younger and older age group respectively) and NMES (1.07 and 0.98 for the younger and older age group respectively).

Analysing both age groups together INTAKE24 provided estimates of energy intake that were just 1% lower on average than the interviewer-led recall with the limits of agreement ranging from minus 49% to plus 93%.

Analysis of the order of allocation of method found no differences in terms of agreement between the two methods. The mean ratio for energy intakes was 0.99 (SD = 1.41) for those completing INTAKE24 first and 1.00 (SD = 1.36) for those completing interviewer-led recall first. The limits of agreement for energy intakes ranged from minus 50% and minus 46% to plus 96% and plus 83% for those completing INTAKE24 first and those completing interviewer-led first respectively. This indicates that reporting of dietary intake using INTAKE24 is as good as the interview-led recall and was not significantly affected by completing the interview first.

Of all the foods and drinks recorded in INTAKE24, 84.9% were an exact match to the food reported in the interviewer-led recall. The most commonly omitted items were drinks (15% of omissions) and vegetables (13%). Breads/cereals made up 15% of omissions, however this was due to a search for ‘cereal’ returning ‘milk on cereal’ in the food list early on in the data collection phase. Participants often selected this thinking it included both the cereal and milk, while it recorded the milk only. This was rectified as soon as the problem was identified.

### 3.3. Agreement between INTAKE24 and Interviewer-Led Recalls, by Food Groups

The results in terms of food groups were varied (Table 4 and Table 5). The foods classified as “Eggs and egg products”, “Vegetables (excluding potatoes)”, and “Nuts and seeds” were reported with reasonable accuracy for both age groups, however the limits of agreement were wide, indicating that the tool worked well at group level only, for these foods. “Cereal & cereal products”, “Fish & fish dishes”, “Cakes, biscuits, pastries, sugar preserves & confectionary” were reported with good accuracy in the 11–16 years old age group, and “Savoury snacks” and “Non-alcoholic beverages” were reported with reasonable accuracy in the older age group.

Intakes of fat spreads tended to be underestimated. This may have been due to individuals forgetting or being unclear on how to multiply the amount of fat spread on bread by the number of slices. This is something the trained interviewer would have asked during the in-person interview. Alcohol intakes also tended to be underestimated. Intakes of alcohol pose particular difficulty due to the effects of alcohol on memory. It may be that verbalising the amount consumed during the in-person interviews, aids recall.

## 4. Discussion

Agreement of INTAKE24 with the reference method compares favourably with relative validation studies of other methods of dietary assessment in both adults and children, although the limits of agreement are slightly wider, particularly for adult participants. Myfood24 also underestimated energy intakes by 3% when compared with interviewer-led recalls in 11–18 years old (*n* = 75), with the limits of agreement ranging from an underestimation of 39% to an overestimation of 34% [14]. Similarly, the computerised 24-h recall system YANA-C (Young adolescents’ nutrition assessment on computer) underestimated energy intakes in 11–14 years old by 3% on average compared to a dietitian-led recall, with the limits of agreement ranging from minus 46% to plus 41% [4] YANA-C was previously tested against a 1-day estimated weighed food diary in the same age group (*n* = 237) and was found to overestimate energy intakes by 13% compared to intakes reported in the diary, with limits of agreement ranging from minus 60% to plus 87% [26]. INTAKE24 performed well in the 11–24 years age group and the results are comparable with the traditional face-to-face interviewer-led recall method, as well as similar computerised methods.

### 4.1. Further Developments

To ensure the INTAKE24 system remains fit for purpose, regular updates will be required to keep food lists and food portion photos up-to-date. Further developments will also be needed to address commonly omitted items such as drinks and vegetables. Although the system already contains prompts to remind users to include drinks, it may be that these need to be more frequent or re-designed so they make it easier for the user to enter forgotten drinks. Refinement of the portion size selection of fat spreads will also be addressed.

Further work is in progress to extend and validate the system for use in adults and older adults and to develop it for use in other ethnic groups and countries. This work includes system developments such as the introduction of a video tutorial and specific help clips, and the facility to capture recipes for home cooked items.

### 4.2. Study Limitations

Practical and financial constraints meant that we were unable to conduct a validation of INTAKE24 against true intake measured by direct meal observation or using objective biomarkers of dietary intake. As both INTAKE24 and the comparison method (interviewer-led recalls) rely on participants’ self-report of food intake, both are prone to bias and true dietary intake is unknown. There are limitations with method comparisons as the act of completing one measure impacts on the accuracy of completion of the other. For example keeping a weighed diary covering the same day as a 24-h recall may improve the accuracy of the recall. Completing a weighed diary covering different days will mean it is impossible to know whether differences are due to the different assessment methods or true variation in dietary intake. For this study we decided to opt for an interviewer-led recall covering the same day and, as the act of completing the first recall is likely to enhance the completion of the second recall, to have the majority of participants (75%) complete the test method (INTAKE24) first. This meant that we were comparing the test method against an interviewer-led recall enhanced by completing INTAKE24 first, allowing us to be as critical as possible of INTAKE24 within the constraints of the study.

Participant recalls were arranged in advance; this may have increased the participants’ awareness of what they were eating the day before. Therefore we acknowledge that this could have impacted on the accuracy of the recalls. However it could be said that this is an advantage of web-based methods compared to in-person methods, as the arrangement of face-to-face appointments is no longer necessary.The target was to recruit 180 participants and for 148 to complete four recall days (allowing 20% drop-out rate). However, due to unforeseen difficulties with the recruitment process resulting in a higher than expected drop-out rate and to ensure the project kept to time, 129 participants completed all four recall days (149 completed at least three recall days).

The study sample was recruited to be representative of the Scottish population, however we acknowledge that the sample has low ethnic diversity.

## 5. Conclusions

The results of the comparison between INTAKE24 with interviewer-led 24-h recalls compare favourably with other method comparison studies of both computerised and face-to-face 24-h recalls.

However commonly forgotten foods were evident. Asking individuals to report their intake prospectively as they go through the day as opposed to recalling intake the following day may reduce the number of forgotten foods and reduce the degree of under-reporting. The development of mobile internet allows users to access the internet “on the go”. INTAKE24 is currently accessible through mobiles and tablet computers and we are aware that some participants used the system on these devices; however we did not record how the tool was accessed in this study. Recent statistics show that 66% of adults in the UK own a smartphone and these are now considered the most important device for accessing the internet among 16–34 years old. The take-up of tablet computers is growing rapidly with over 54% of households now owning at least one [27]. Therefore a tool which enables the user to record throughout the day warrants exploration.

Dietary assessment methods that utilise technology may be more appealing and engaging than paper based methods, particularly for children and young adults. Web-based methods can be conducted at a time and place convenient to the participant, without the need for an in depth face-to-face interview. Online methods can be deployed to large population groups with minimal impact on resource compared with methods requiring in-field researchers. The nature of web-based tools allows for greater coverage of a population group, improving the representativeness of the sample captured. They also have the benefit of ensuring standardisation of methods, as the quality of the data collected and the accuracy of food coding and data entry do not vary with the experience and diligence of the dietitian or researcher. Nutrient output may be available as soon as the participant has completed their recall making instant feedback possible with potential for application as an intervention as well as an assessment tool. System developments are ongoing, and further work is in progress to improve and develop INTAKE24 for use in other populations.

## Figures and Tables

**Table 1:** Participant demographics (all participants completing at least one corresponding INTAKE24 and interviewer-led recall; *n* = 168).

		11–16 Year-Old	17–24 Year-Old
*n*		52	116
Gender	Male	19 (36%)	55 (47%)
	Female	33 (64%)	61 (53%)
BMI ^a^ (SD)		22.1 (4.9)	23.7 (3.5)
Economic Status	Higher Education	0 (0%)	42 (36%)
	Looking after family/home	0 (0%)	3 (3%)
	School	52 (100%)	11 (10%)
	Unemployed	0 (0%)	13 (11%)
	Working	0 (0%)	47 (40%)
Ethnicity	White	49 (94%)	109 (94%)
	Non-white	3 (6%)	7 (6%)

^a^ height and weight data available for 97 participants (35 11–16 years old; 62 17–24 years old).

**Table 2:** Agreement of intakes reported using INTAKE24 with interviewer-led recalls for 11–16 years old (participants completing any number of days, *n* = 52).

	INTAKE24 Geometric Mean	Interview Geometric Mean	Ratio of Geometric Mean	Limits of Agreement	
				Lower	Upper
Energy (kJ)	6681.7	6823.9	0.97	0.52	1.82
Carbohydrate (g)	234.2	236.0	0.99	0.52	1.88
NSP (g)	9.1	9.6	0.94	0.45	1.98
Fat (g)	52.3	55.8	0.92	0.43	1.96
Fat (%)	29.5	31.0	0.95	0.63	1.42
Saturated Fat (g)	19.2	20.5	0.92	0.39	2.17
Protein (g)	52.4	52.4	0.99	0.47	2.11
NMES (g)	89.3	85.3	1.07	0.28	4.05
NMES (%)	22.4	20.8	1.10	0.32	3.75
Alcohol (g)	0.01	0.01	0.95	0.29	3.10
Vitamin C (mg)	104.3	96.7	1.09	0.44	2.71
Iron (mg)	8.1	8.3	0.98	0.45	2.11
Calcium (mg)	713.9	705.8	1.00	0.46	2.18

NSP (non-starch polysaccharides); NMES (non-milk extrinsic sugars).

**Table 3:** Agreement of intakes reported using INTAKE24 with interviewer-led recalls for 17–24 years old (participants completing any number of days, *n* = 116).

	INTAKE24 Geometric Mean	Interview Geometric Mean	Ratio of Geometric Mean	Limits of Agreement	
				Lower	Upper
Energy (kJ)	7408.1	7515.5	1.00	0.50	1.98
Carbohydrate (g)	229.1	230.3	1.02	0.52	2.00
NSP (g) ^a^	11.4	11.3	1.02	0.46	2.27
Fat (g)	63.1	62.7	0.99	0.43	2.32
Fat (%)	31.7	31.8	1.00	0.58	1.70
Saturated Fat (g)	22.5	22.3	0.99	0.38	2.62
Protein (g)	64.2	62.9	1.02	0.42	2.51
NMES (g) ^b^	60.6	62.3	0.98	0.22	4.39
NMES (%) ^b^	13.9	13.7	0.98	0.23	4.25
Alcohol (g)	0.12	0.15	0.87	0.08	9.79
Vitamin C (mg)	74.8	73.1	1.03	0.18	5.81
Iron (mg)	9.1	9.3	0.99	0.44	2.25
Calcium (mg)	726.6	716.2	1.03	0.42	2.54

^a^ non-starch polysaccharides; ^b^ non-milk extrinsic sugars.

**Table 4:** Agreement of intakes of main food groups for 11–16 years old, (*n* = 52).

	INTAKE24 Geometric Mean	Interview Geometric Mean	Mean Ratio	Limits of Agreement	
				Lower	Upper
Cereals and cereal products	70.71	72.67	0.97	0.47	3.08
Starchy carbohydrates	137.49	114.68	1.20	0.50	3.61
Milk and milk products	161.95	120.32	1.35	0.46	1.96
Eggs and egg products	1.69	1.78	0.95	0.41	1.97
Fat spreads	2.87	3.19	0.90	0.27	4.43
Meat and meat products	67.73	62.52	1.08	0.23	4.09
Fish and fish dishes	3.32	3.43	0.97	1.00	1.00
Vegetables (excluding potatoes)	11.18	11.22	1.00	0.26	3.07
Savoury snacks	12.46	13.92	0.90	0.61	1.63
Nuts and seeds	1.02	1.03	0.99	0.18	7.32
Fruit	92.59	79.71	1.16	0.38	2.65
Cakes, biscuits, pastries, sugar preserves and confectionary	32.86	32.96	1.00	0.18	7.33
Non-alcoholic beverages	696.19	605.31	1.15	0.57	1.63
Alcoholic beverages	1.06	1.10	0.96	0.10	12.44
Miscellaneous	8.81	7.83	1.12	1.00	1.00

**Table 5:** Agreement of intakes of main food groups for 17–24 years old (*n* = 116).

	INTAKE24 Geometric Mean	Interview Geometric Mean	Mean Ratio	Limits of Agreement	
				Lower	Upper
Cereals and cereal products	75.45	82.39	0.92	0.52	2.42
Starchy carbohydrates	145.90	130.73	1.12	0.28	5.28
Milk and milk products	78.89	64.60	1.22	0.33	2.90
Eggs and egg products	2.77	2.85	0.97	0.18	3.57
Fat spreads	2.45	3.08	0.79	0.44	2.62
Meat and meat products	71.80	67.09	1.07	0.28	4.15
Fish and fish dishes	4.24	3.93	1.08	0.80	1.27
Vegetables (excluding potatoes)	27.05	25.67	1.05	0.36	2.68
Savoury snacks	5.99	6.12	0.98	0.43	2.21
Nuts and seeds	1.90	1.94	0.98	0.25	4.40
Fruit	64.16	61.54	1.04	0.38	3.38
Cakes, biscuits, pastries, sugar preserves and confectionary	35.88	31.79	1.13	0.56	1.90
Non-alcoholic beverages	1175.44	1134.30	1.04	0.20	4.04
Alcoholic beverages	5.73	6.31	0.91	0.14	9.63
Miscellaneous	14.60	12.68	1.15	1.00	1.00
